# Validity of the KOJI AWARENESS self-screening test for body movement and comparison with functional movement screening

**DOI:** 10.1371/journal.pone.0277167

**Published:** 2022-12-30

**Authors:** Koji Murofushi, Daisuke Yamaguchi, Hiroki Katagiri, Kenji Hirohata, Hidetaka Furuya, Sho Mitomo, Tomoki Oshikawa, Koji Kaneoka, Hideyuki Koga, Kazuyoshi Yagishita

**Affiliations:** 1 Sports Science Center, Tokyo Medical and Dental University (TMDU), Tokyo, Japan; 2 Japan Sports Agency, Tokyo, Japan; 3 Department of Joint Surgery and Sports Medicine, Graduate School of Medical and Dental Sciences, Tokyo Medical and Dental University (TMDU), Tokyo, Japan; 4 Department of Orthopedics, Dokkyo Medical University Saitama Medical Center, Saitama, Japan; 5 Clinical Center for Sports Medicine and Sports Dentistry, Tokyo Medical and Dental University (TMDU), Tokyo, Japan; 6 Department of Rehabilitation, Sonoda Third Hospital/Tokyo Medical Institute Tokyo Spine Center, Tokyo Japan; 7 Faculty of Sport Science, Waseda University, Tokyo, Japan; JAPAN

## Abstract

**Objective:**

This study aimed to validate the KOJI AWARENESS™, a self-screening movement test, and compare it with the Functional Movement Screen (FMS).

**Methods:**

Fifty-seven healthy participants completed the KOJI AWARENESS™ and functional movement screening. Pearson’s correlation coefficients were used to assess the validity of the test. Subsequently, partial correlation analysis was used to determine the associations between age, sex, body mass index, and sports level as control variables and motor function.

**Results:**

Correlation and partial correlation analyses showed a strong positive correlation between the functional movement screening and the KOJI AWARENESS™ scores.

**Conclusion:**

This study found that the KOJI AWARENESS™ test is valid and comparable to functional movement screening. It can be used for self-screening of movement.

## Introduction

Currently, individuals can monitor various aspects of their health status, such as the quality of sleep and body composition, through tools available for self-checks, which help maintain health status [1]. Self-assessment tools for various parts of body mobility and stability have been developed [2]. As a result, health-conscious people use these to prevent diseases and physical problems.

Various musculoskeletal screening and functional performance tests have been conducted in medical, healthcare, and sports settings to assess the physical conditions of individuals. Assessments of gait, static, and dynamic balance play an important role in monitoring and guarding the health of older adults [3–6]. Numerous previous reports have shown that physical function problems, such as muscle weakness and limited range of motion, are associated with the incidence of pain, sports injuries, and trauma [7–10]. Consequently, musculoskeletal screening tests for injury prevention have been widely used in medical and sports settings.

The Functional Movement Screen (FMS) was developed to monitor movement patterns, including stability and mobility in extremities, which may enable a conditioning coach to monitor those who fail to develop strength, speed, and power [11,12]. A systematic review of the FMS showed intraobserver reliability of 0.81 (95% confidence interval [CI], 0.69–0.92) and interobserver reliability of 0.81 (95% CI, 0.70–0.92), indicating that the FMS had excellent reproducibility [13]. In addition, a review of nine studies on the predictive value of the FMS score showed that participants with FMS scores of ≤14 were 2.74 times more likely to experience injury during activity than those with higher scores [13]. FMS has been widely utilized in sports rehabilitation, sports science, and possibly sports injury prevention [14–17].

Because the FMS requires the professionals who completed their certification workshop to implement, other individuals cannot use it without such skilled knowledge of functional anatomy, such as planes of motion, to assess movement patterns. For example, the FMS allows the trained professionals to simultaneously evaluate multiple joint movement patterns, such as performing squat, which requires the evaluator for the need of experience to perform the evaluation; otherwise, it may result in poor reproducibility [18,19]. Thus, it is imperative that an alternative tool that allows health-conscious individuals, including older adults and athletes, to evaluate specific movements without equipment or experts needs to be developed. However, there is no availability of such a self-administrated motor functional screening test or scoring system that may identify the individuals who may be vulnerable to injury or who may not be ready to compete in sports.

Murofushi et al. developed a self-screening test called KOJI AWARENESS™. There was a significant negative correlation between the KOJI AWARENESS™ score and the grade of musculotendinous soreness for those who were regularly trained [20]. Furthermore, there are a number of similarities between KOJI AWARENESS™ and the FMS [21,22], such as the range of motion measurements [22–27] and muscle strength measurements [28–32]. Therefore, the KOJI AWARENESS^TM^ is a tool that can be active in self-screening and predicted to be comparable to the FMS score.

The present study aimed to analyze the KOJI AWARENESS™, a self-screening movement test score, and compare it with the FMS score to determine whether KOJI AWARENESS™ can be practically used as a self-screening movement test. We hypothesized that the KOJI AWARENESS™ score would be positively correlated with the FMS score, which was used for external validation. In addition, the KOJI AWARENESS™ is comparable to the FMS and can be alternatively used for movement self-screening.

## Materials and methods

### Participants

This study was conducted at the fitness centers of the authors’ affiliated institutions under the guidance of the Department of Joint Surgery and Sports Medicine, Graduate School (October 2019-March 2020). It was approved by the Research Ethics Committee of the authors’ affiliated institutions (research protocol identification number: M2019-168). The individual pictured in Appendix has provided written informed consent (as outlined in PLOS consent form) to publish their image alongside the manuscript. The total number of participants included in this study was 57. All the participants provided written informed consent for participation in this study before the screening. All the participants completed the KOJI AWARENESS™ and FMS on the same day. The tests were randomly administered with an adequate rest between tests to minimize the effect of fatigue. The participants wore comfortable clothes that allowed for athletic movements.

Before the measurement, all participants provided their physical attributes (height, weight, sex, and age), medical history, sports history, sports levels, and daily activity questionnaire for analysis. We defined participants as athletes if they were actively participating in competitive sports and non-athletes if they were not. Any of the participants were excluded if they were unable to participate in their sports activity due to injuries more than one month within the last three months. The participants were instructed to stop when they felt pain during any part of the test. However, none of the participants discontinued the study due to injury or pain throughout the study.

### The KOJI AWARENESS^TM^ self-screening movement test

Further details on the KOJI AWARENESS^TM^ self-screening movement test are provided in S1 and S2 Appendices. Athletes use a checklist to self-evaluate the function of each body part and have 11 components of the test. Each component has distinct scoring criteria, with a maximum total score of 50 points. The self-scoring method was explained to the participants. It was confirmed verbally that the subjects fully understood the self-scoring method. Subsequently, they self-rated the motor function of each item according to the method presented (S1 and S2 Appendices). For this test, up to three attempts were allowed, and the best score was retained. All the exercises demonstrated by the participants were photo-documented to ensure accurate scoring. The participants completed the assessment within an average of 20 min.

Before this study, a pilot test was conducted to examine the reproducibility of KOJI AWARENESS™. Ten participants were invited to assess the reproducibility of the KOJI AWARENESS™ approximately seven days after their first administration. The intra-observer reliability of the KOJI AWARENESS™ was assessed using the intraclass correlation coefficients (ICCs). The ICC (1,1) for the intraobserver reliability of the KOJI AWARENESS™ was 0.876 (95% CI, 0.434–0.981), and its high reproducibility was confirmed.

### FMS

The FMS has been extensively described previously [11–13,17]. The following seven screening tests are used to evaluate different movement patterns: deep squat, hurdle step, in-line lunge, shoulder mobility, active straight leg raise, trunk stability, and push-up and rotary stability. Three of these are clearing tests for determining the pain response. Each test has a possible score of 3 points and a maximum of 21 points. All participants were assessed by an athletic trainer certified by the Board of Certification, Inc and for the FMS. Up to three attempts were allowed for this test, and the best score was retained. If pain was produced from three clearing tests, a score of 0 was assigned to each of the three categories.

### Patient and public involvement

No patients were involved in setting the research question or the outcome measures, developing plans, or implementing the study. No patients were asked for advice on interpreting or writing the results.

### Statistical analyses

The normality of the distribution of each variable was confirmed using histograms and the Shapiro–Wilk test. The mean ± standard deviation was used to summarize the normally distributed data, and the median (interquartile range) was used for data that were not normally distributed.

At first, a correlation analysis was conducted to determine whether the KOJI AWARENESS^TM^ score was related to the FMS score. Then, a partial correlation analysis was conducted for each control variable, using sex, age, BMI, and sport level as control variables that could be related to motor function. Finally, a partial correlation analysis was conducted with all control variables simultaneously. The correlation was considered ‘‘strong” (r > 0.5), ‘‘medium” (0.5 < r < 0.3), or ‘‘weak” (0.3 < r < 0.1) [33]. *P*-values <0.05 were considered statistically significant. The data were analyzed using SPSS (version 21.0; IBM Corp, Armonk, NY, USA).

## Results

The mean age of the participants was 31.7 ± 9.6 years, and their mean BMI was 23.3 ± 4.9 kg/m^2^. Twenty-nine (50.9%) were female, and 22 (38.6%) were classified as athletes (Table 1). Table 1 also presents the demographic data by sex. The average age and BMI for females were 29.4±9.3 years and 20.7± 2.0 kg/m^2^, respectively, while the average age and BMI for males were 34.1±9.5 years and 25.9±5.5 kg/m^2^, respectively (Table 1).

**Table 1:** Demographic data (N = 57).

	All	Female (n = 29)	Male (n = 28)
Age, y	31.7 ± 9.6	29.4 ± 9.3	34.1 ± 9.5
Female: Male, n (%)	29 (50.9): 28 (49.1)	-	-
Body mass index, kg/m^2^	23.3 ± 4.9	20.7 ± 2.0	25.9 ± 5.5
Sports Level, Athlete: Non-athlete, n (%)	22 (38.9): 35 (61.1)	13 (44.8): 16 (55.2)	9 (32.1): 19 (67.9)

**Fig 1:**
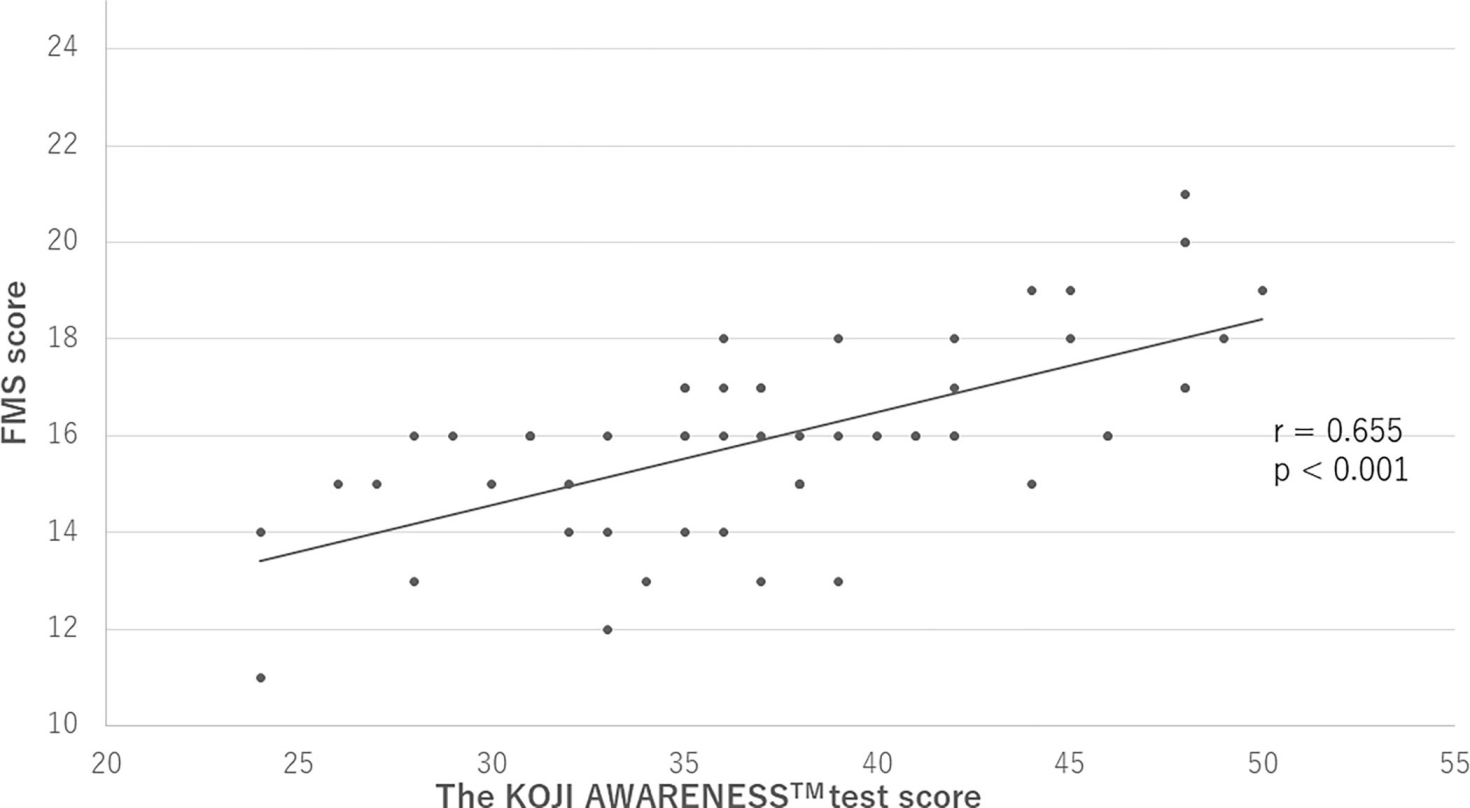
Scatter plot of the FMS score vs. the KOJI AWARENESS^TM^ score (N = 57). FMS, Functional movement screening.

The KOJI AWARENESS^TM^ and FMS average scores were 37.6 ± 6.7 and 16.0 ± 2.0, respectively (Table 2). A scatter plot showing the relationship between the FMS and KOJI AWARENESS™ scores is shown in Fig 1. The correlation analysis showed a strong positive correlation (r = 0.655, p < 0.001) between the FMS and KOJI AWARENESS™ scores (Table 3). Partial correlation analysis, in which control variables (age, sex, BMI, and sport level) that may affect motor function were put one at a time, showed a significant positive correlation between the FMS and KOJI AWARENESS™ scores in each (Table 3). Furthermore, a partial correlation analysis, in which all control variables (age, sex, BMI, and sport level) that could affect motor function were entered, found a significant positive correlation between the FMS and KOJI AWARENESS™ scores (r = 0.576, p < 0.001, Table 3). There was no difference in the correlation coefficients from the correlation analysis, the partial correlation coefficients for each control variable, and the partial correlation coefficients with all control variables simultaneously entered (Table 3).

**Table 2:** Outcome scores in each test (N = 57) ^α^.

Outcome	Score
FMS score	16.0 ± 2.0
KOJI AWARENESS score	37.6 ± 6.7

^α^ Data are reported as mean± standard deviation.

FMS, Functional movement screening.

**Table 3:** Correlation and partial correlation between the FMS and KOJI AWARENESS scores (N = 57).

^ ^	FMS score		Control variables
	r-value	P-value	
KOJI AWARENESS^TM^ ^α^	0.655	< 0.001	None
KOJI AWARENESS^TM^ ^β^	0.630	< 0.001	Sex
KOJI AWARENESS^TM^ ^β^	0.609	< 0.001	Age
KOJI AWARENESS^TM^ ^β^	0.655	< 0.001	BMI
KOJI AWARENESS^TM^ ^β^	0.619	< 0.001	Sports Level
KOJI AWARENESS^TM^ ^β^	0.576	< 0.001	Sex, age, BMI, sports level

^α^ Results from Pearson’s correlation analysis.

^β^ Results from partial correlation analysis.

FMS, Functional movement screening; BMI, body mass index.

## Discussion

This study analyzed the self-evaluation test, KOJI AWARENESS™, compared its score with the FMS score, and investigated whether KOJI AWARENESS™ can be alternatively used as a self-screening movement test. The results show a strong correlation between the scores of the two tests (Table 3). There was also a significant correlation between the scores of the two tests when age, sex, BMI, and sports level were analyzed as control variables (Table 3).

Loudon et al. [17] examined the FMS scores for runners and found that female runners scored higher on each test than male runners. Furthermore, younger runners scored higher than older runners [17]. Perry et al. [15] revealed that older age and BMI were associated with lower FMS scores. These previous reports suggest that demographic characteristics may influence the movement pattern of individuals. Therefore, the validity of the KOJI AWARENESS™ test and its comparability to the FMS were analyzed using partial correlation analysis, and the influence of the demographic characteristics and the levels of competition were assessed. In the present study, the KOJI AWARENESS™ and FMS scores were positively correlated, even after accounting for age, sex, and competition level. From the above, we suggest that the KOJI AWARENESS™, which was assessed by the participants, has external validity for evaluating motor function without a trained examiner.

The results of the current study suggest that the KOJI AWARENESS™ is effective in assessing body movement. The scores may help develop individual conditioning plans to improve movement function. Self-monitoring of daily habits effectively improves adherence to good exercise and diet behaviors [34,35]. Compared to FMS, KOJI AWARENESS™ is highly versatile in that the subjects can easily evaluate themselves without needing a physiotherapist or trainer with specialized knowledge. Therefore, the KOJI AWARENESS™ may be useful as a self-monitoring tool for movement functions and help people establish and improve their movement habits.

Our study has several limitations. First, because the measurements in this study were taken at a single center, there may be selection bias. It is unclear whether the results of this study are generalizable. Second, this study did not include people with pain or symptoms associated with impairment or injury. If patients with impairment or injury are included, the scores may change and affect the results. Third, since this study involved young, healthy participants, it is unclear whether it applies to middle-aged and older adults. Finally, in this study, the FMS was used as an external reference to confirm the validity of the KOJI AWARENESS™. There is no consensus on the validity of the FMS-based prediction of sports injuries [13]. In this study, the association between FMS and KOJI AWARENESS™ was analyzed only in terms of correlation and not in terms of association with sports injury risk. Therefore, the association between KOJI AWARENESS™ and sports injury risk cannot be mentioned, and it is unclear whether it is an effective alternative to FMS. Future studies should involve a large sample of participants, including youth, middle-aged, older adults, and patients, and prospective cohort studies should be designed to analyze the association between the KOJI AWARENESS™ score and the risk of sports injuries.

## Conclusions

The results of this study showed that the self-screening movement test, also known as the KOJI AWARENESS™, was comparable to the FMS. Therefore, it can be effectively used for self-assessments of the movements of healthy individuals.

## Supporting information

S1 File (PDF)

S1 Appendix 11-component movement test. (PDF)

S2 Appendix Scoring criteria. (PDF)
