# Supplementary material for: Validity of the KOJI AWARENESS self-screening test for body movement and comparison with functional movement screening
Source: PLoS One. 2022 Dec 30;17(12):e0277167. doi: 10.1371/journal.pone.0277167 (PMC9803145; doi:10.1371/journal.pone.0277167)
Supplement: S1 File — (PDF) [file pone.0277167.s001.pdf]

PLOS

Consent Form for Publication in a PLOS Journal

I, the undersigned, give my consent for my or my minor child's (insert name below, where indicated) photograph, other image or likeness, case history or family history to be published in a Public Library of Science (PLOS) Journal. I have seen and read the material to be published. I have discussed this consent form with Hiroki Katagiri, who is an author of this article, and I understand the following:

All PLOS journals are freely available on the web[1]. Hence, anyone anywhere in the world can read material published in them. Readers include not only doctors, but also journalists and other members of the public.

I understand and acknowledge each of the following:

While my name will not be published and PLOS will attempt to remove any information that could identify me, it is not possible to ensure complete anonymity, and someone may nevertheless be able to recognize me.

The text of the article may be edited for style, grammar, consistency, and length in the course of the review process.

Under the license which PLOS uses (the Creative Commons Attribution License[2]) material published in PLOS journals can be redistributed freely and used for any legal purpose, including translation into other languages and commercial uses. I understand that I will not receive payment or royalties for this material, and I do not have a claim on any possible future commercial uses of this content.

Signing this consent form does not remove my rights to privacy. I may revoke my consent at any time before publication, but once the information has been committed to publication ("gone to press"), revocation of the consent is no longer possible.

If other family members are referenced (eg in a family history) I confirm I have their consent also to publication.

Name Koji Murofushi

Name of minor child if this Consent Form pertains to them: ____________________

Date 15/11/2022

Signed 室伏 広治

Author Kaji Murofushi

Date 15/11/2022

Signed 室伏 広治

PLOS Patient Consent Form

[1]PLOS Journals: https://www.plos.org/publications

[2]CC-BY; for current and prior versions, see https://creativecommons.org/licenses/

Please complete this form, obtain the patient's signature, and file in case notes.

The manuscript reporting this patient's details should state that consent to publication was obtained from the patient.
