# Supplementary material for: Validity of the KOJI AWARENESS self-screening test for body movement and comparison with functional movement screening
Source: PLoS One. 2022 Dec 30;17(12):e0277167. doi: 10.1371/journal.pone.0277167 (PMC9803145; doi:10.1371/journal.pone.0277167)
Supplement: S1 Appendix — (PDF) [file pone.0277167.s002.pdf]

S1 Appendix

KOJI AWARENES™

S1 Appendix

KOJI AWARENESS™ movement test

1, Neck Mobility

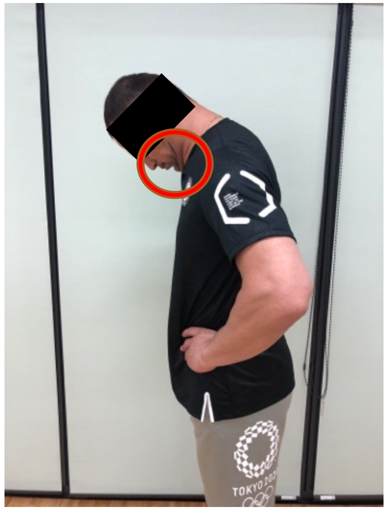

1 point

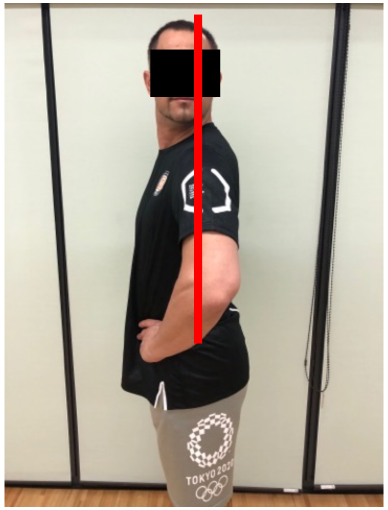

L: 1 point, R: 1 point

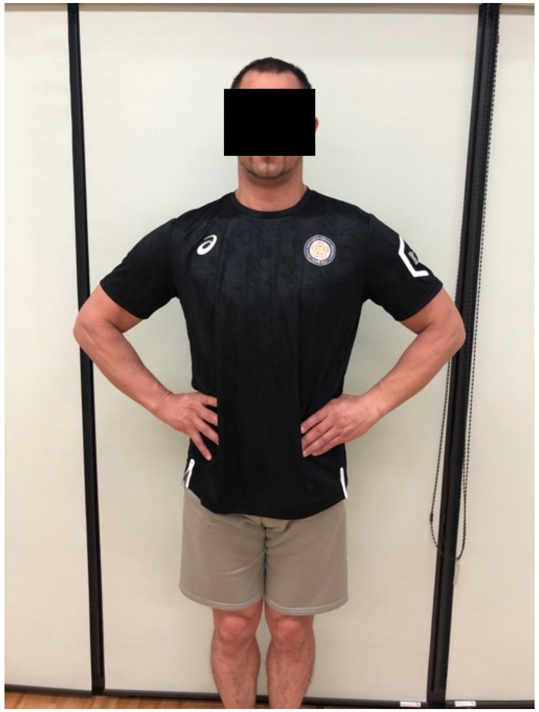

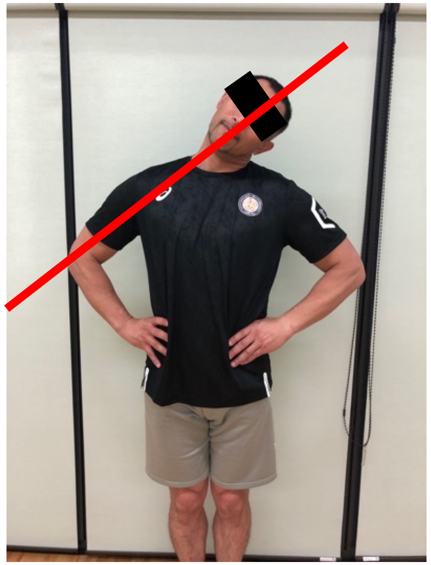

L: 1 point, R: 1 point

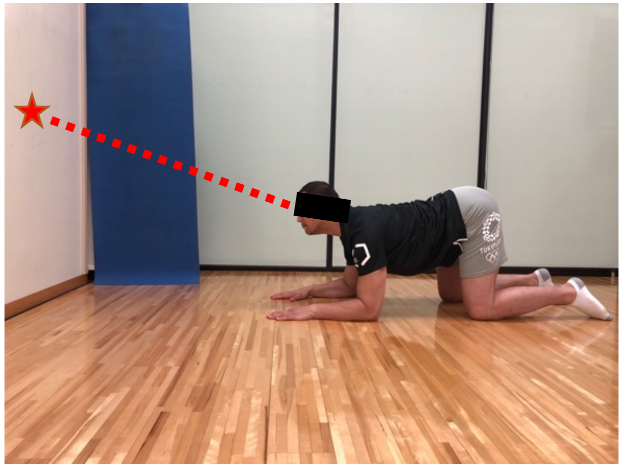

1 point

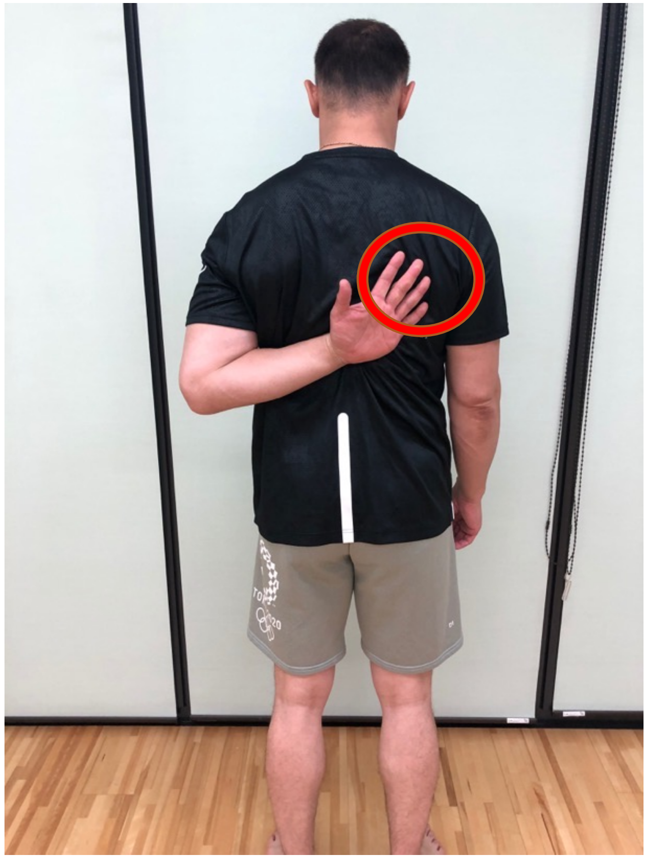

2, Shoulder Mobility

L: 1 point, R: 1 point

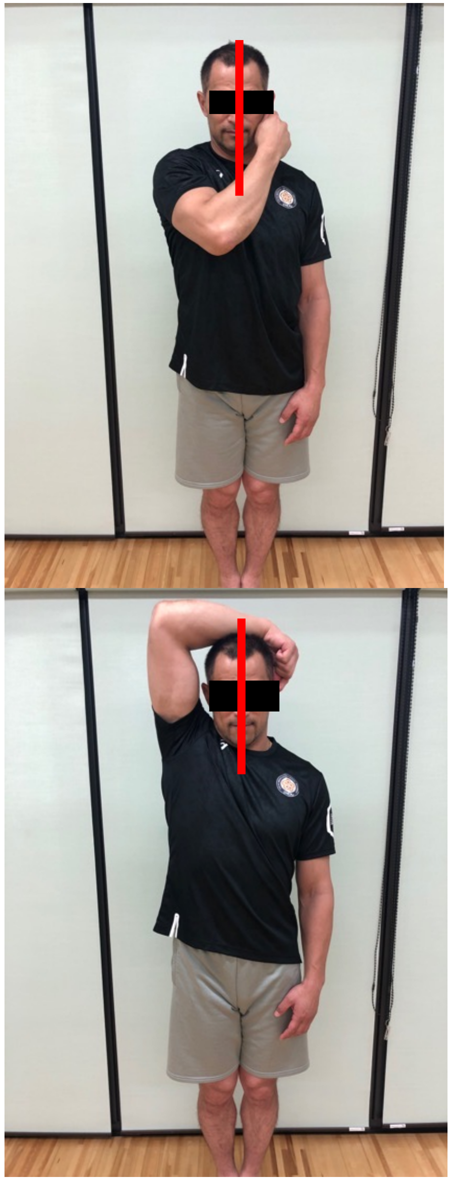

3, Shoulder Blade (Scapular)Mobility

L: 1 point, R: 1 point

4, Thoracic Spine Mobility

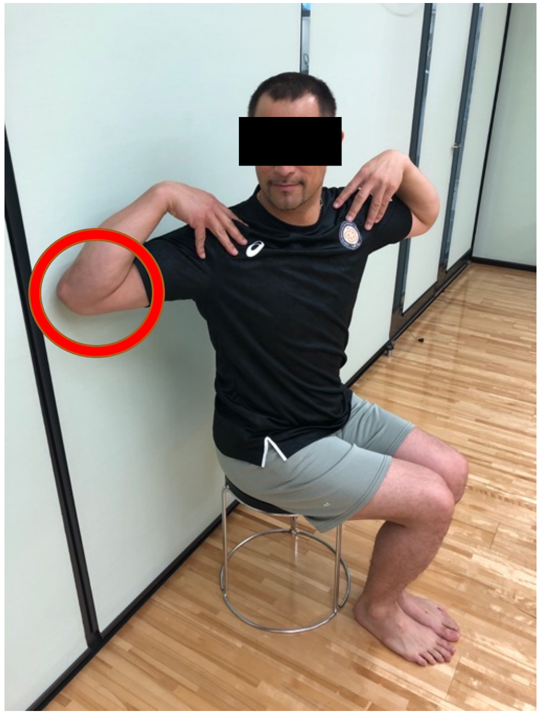

1 point

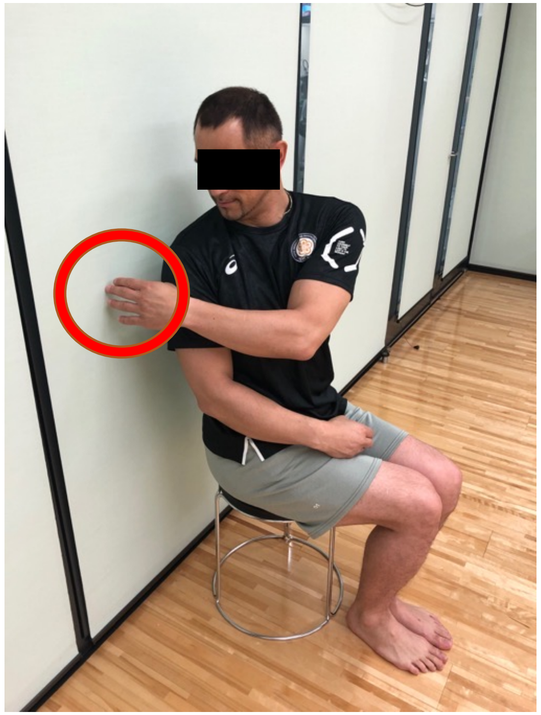

2 point

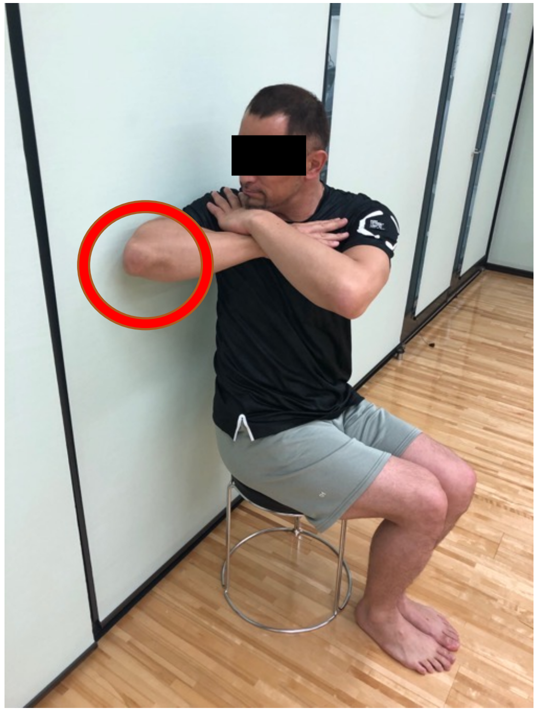

3 point

5, Upper Extremity Stability & Strength

10 sec

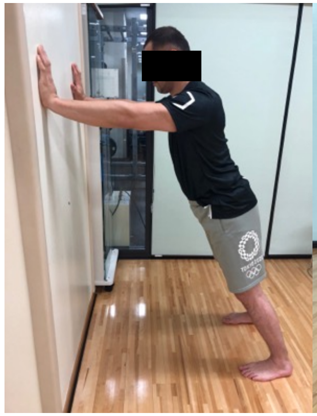

1 point

10 sec

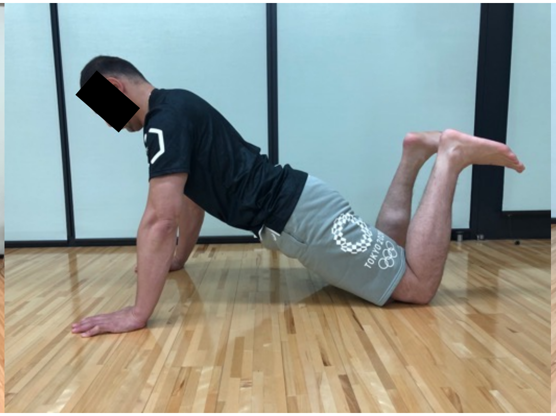

2 point

10 sec

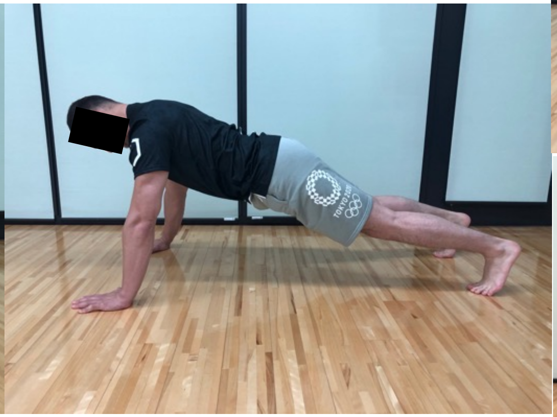

3 point

3 sec

5 sec

3 sec

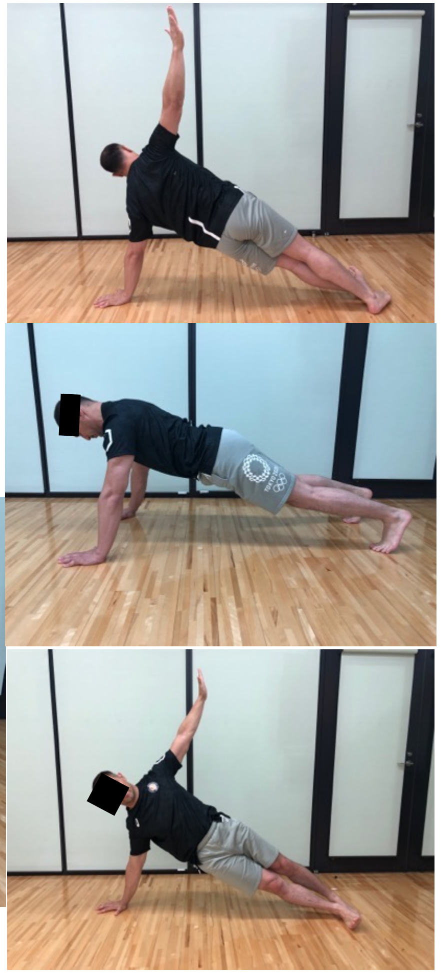

4 point

6, Hip Mobility

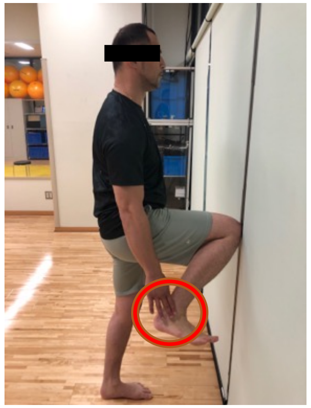

L: 1 point, R: 1 point

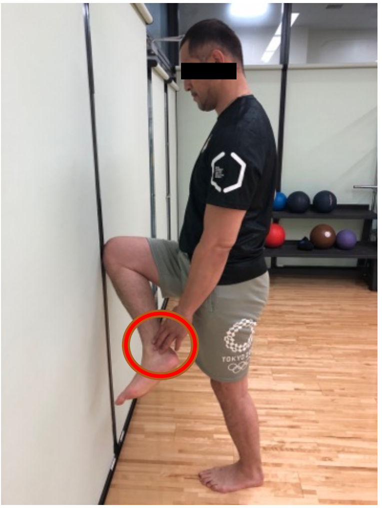

L: 1 point, R: 1 point

Flex Internal and External Rotation

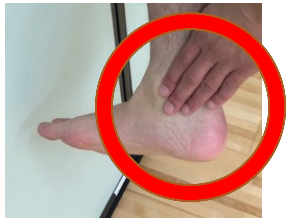

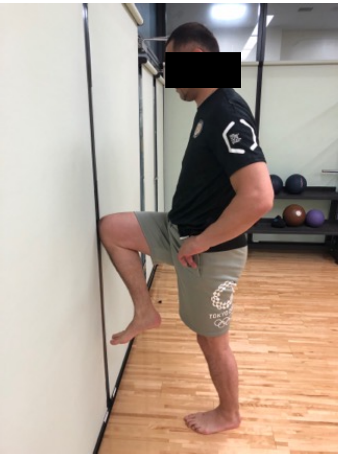

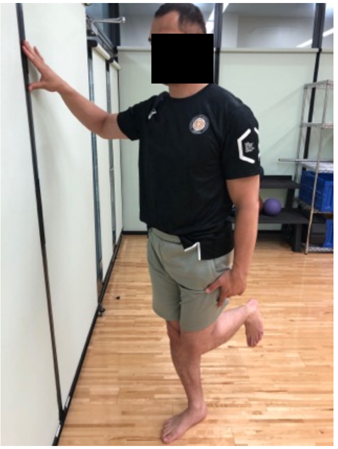

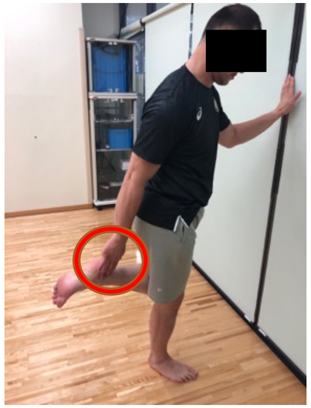

L: 1 point, R: 1 point

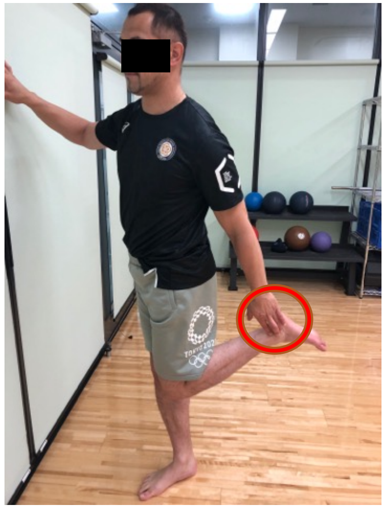

L: 1 point, R: 1 point

Extend Internal and External Rotation

7, Hip and Spine Mobility

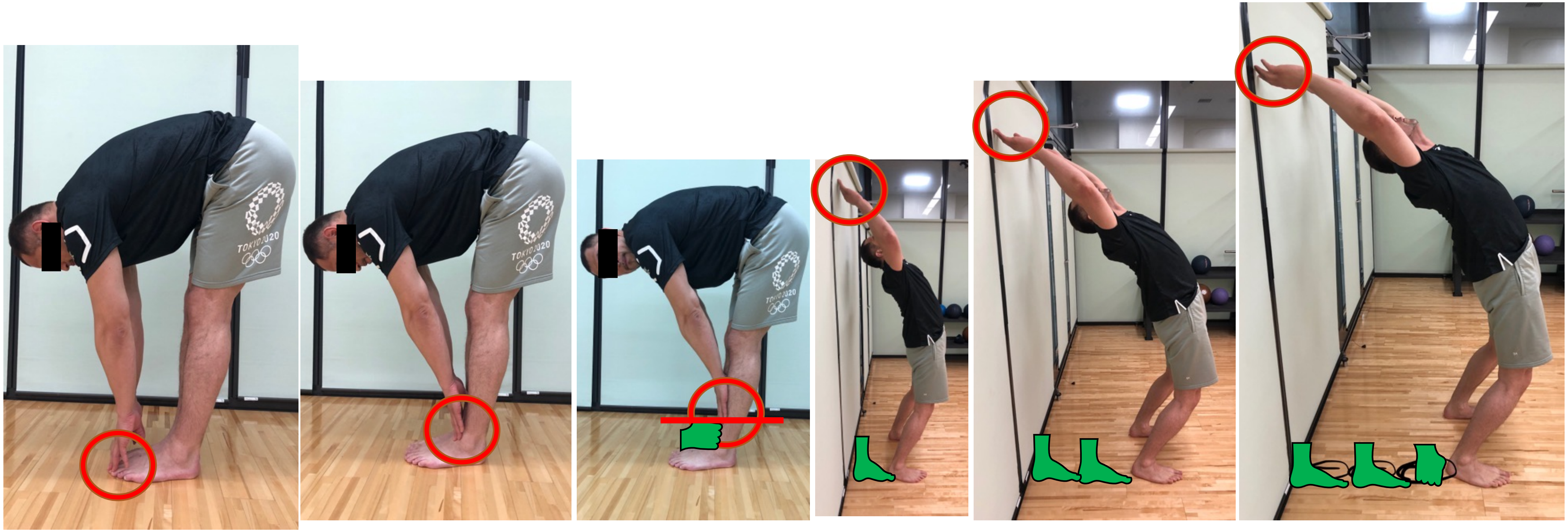

3 point | 2 point | 1 point | 1 point | 2 point | 3 point

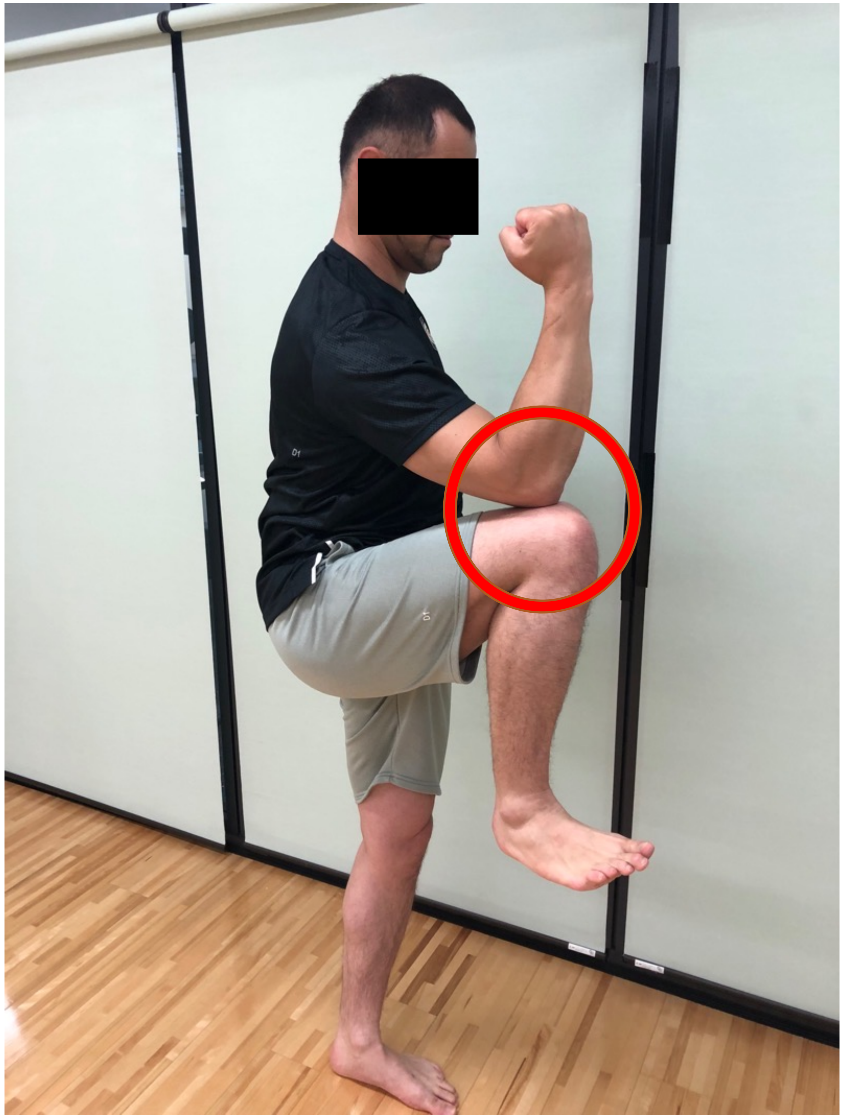

3 sec L: 1 point, R: 1 point

8, Upper and Lower Extremity Mobility and Stability

Clearing Test

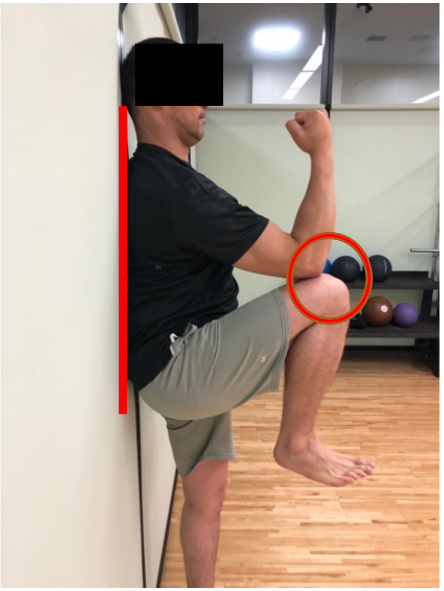

9, Mid-section Stability Strength

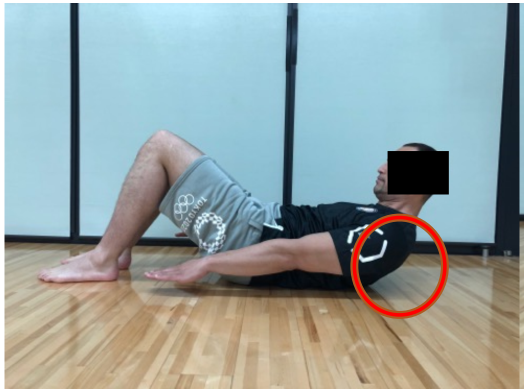

1 point

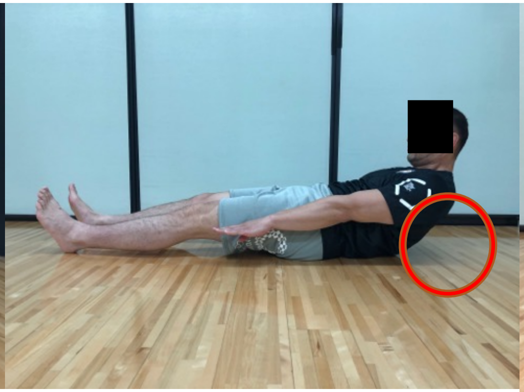

2 point

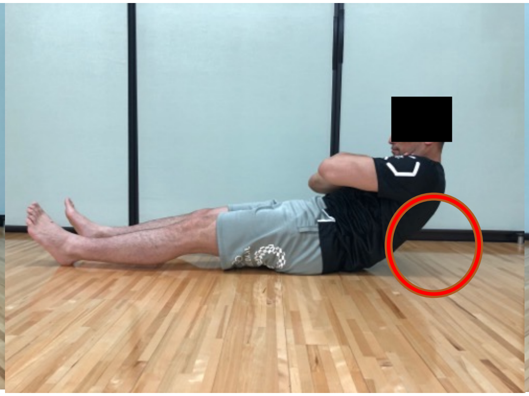

3 point

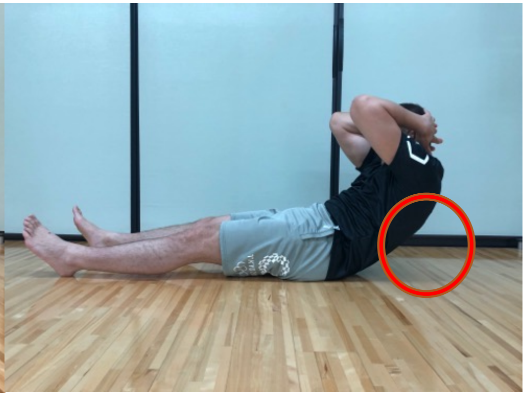

4 point

10, Lower Extremity Strength

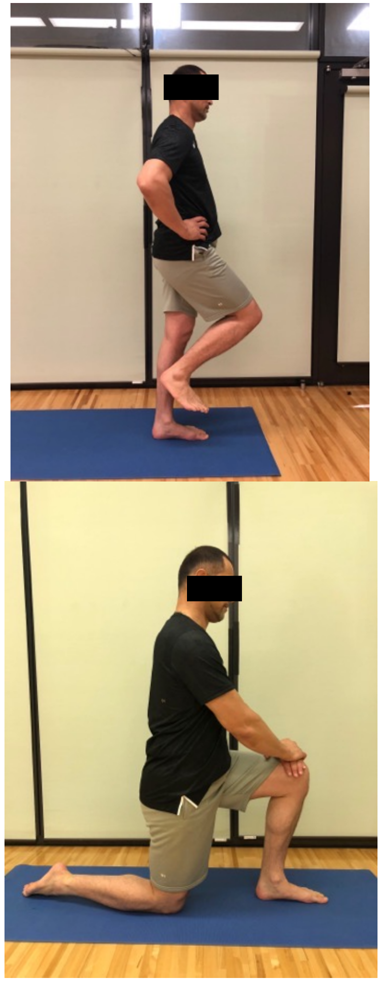

1 point

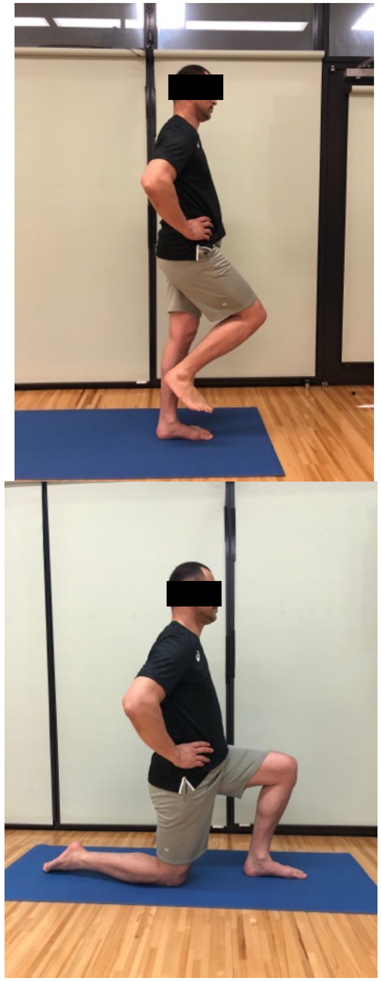

2 point

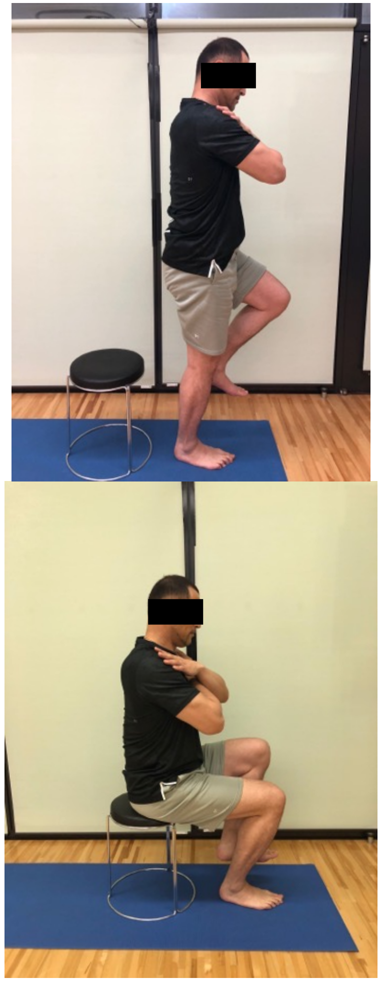

3 point

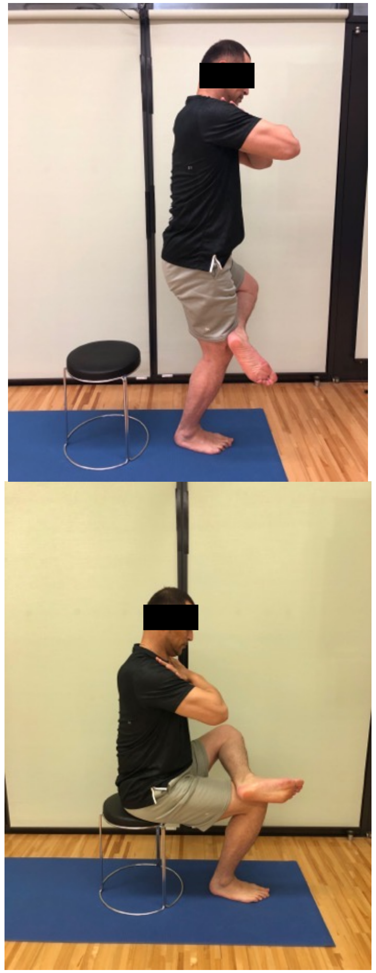

4 point

11, Ankle Mobility

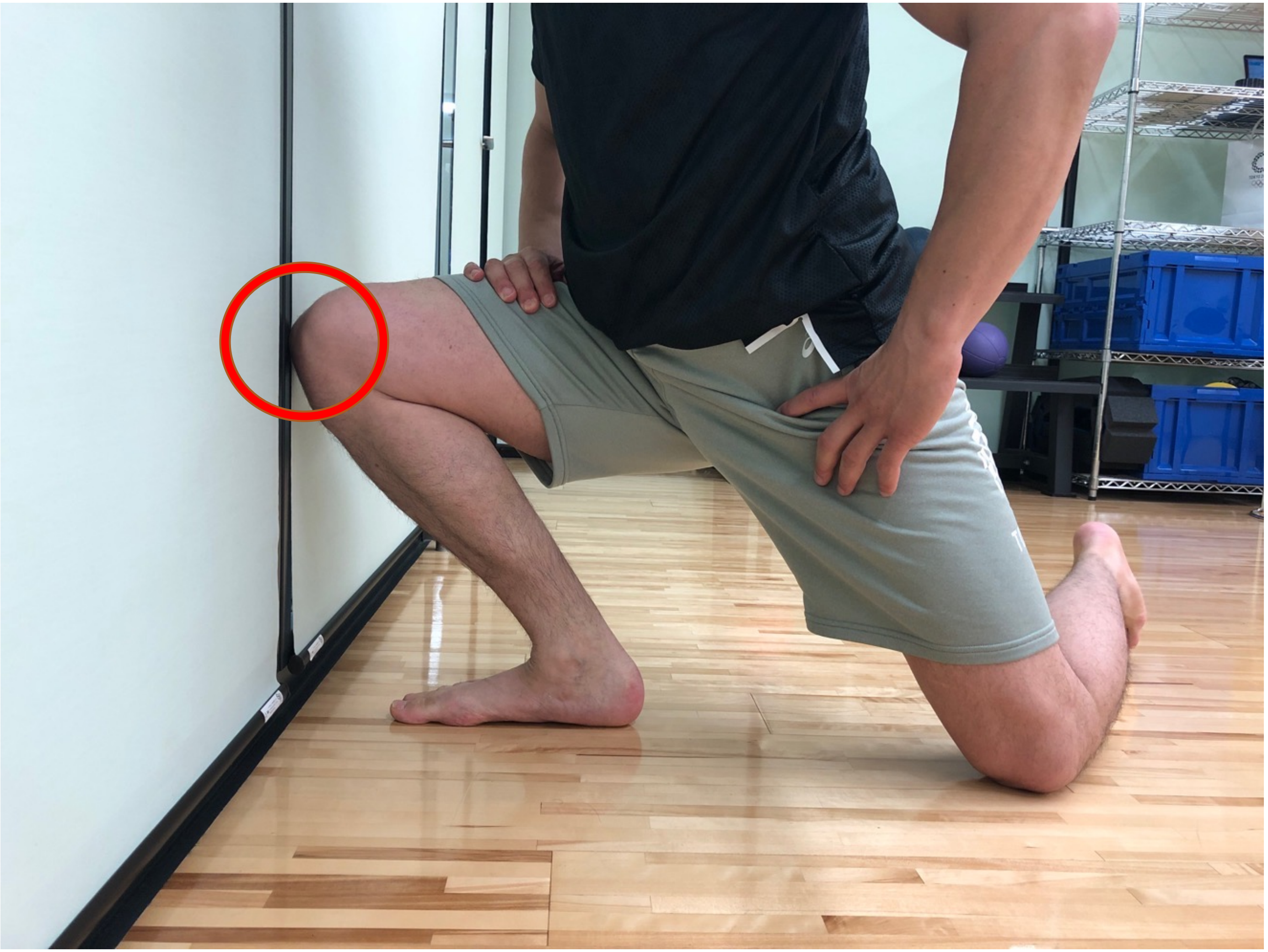

L: 1 point, R: 1 point

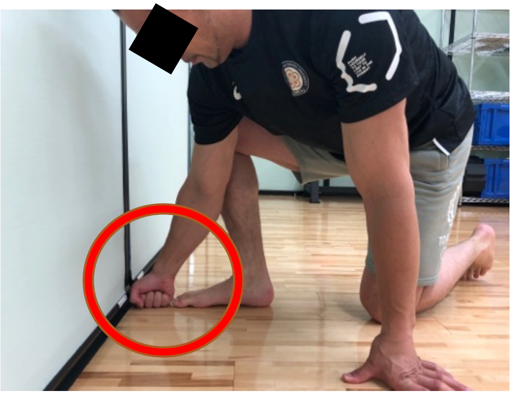

Scoring Chart Sheet

Name（sports, organization, record, gender, DOB）			DATE	1	2	3	4
KOJI AWARENESS			TOTAL	SCORE	SCORE	SCORE	SCORE
Check 1: Neck Mobility （Total 6 Point）		Front	1				
		Back	1				
		Left	1				
		Right	1				
		Right Rotation	1				
		Right Rotation	1				
Check 2: Shoulder Joint Mobility （Total 2 Point）		Left	1				
		Right	1				
Check 3: Scapular Mobility （Total 2 Point）		Left	1				
		Right	1				
Check 4: Thoracic Spine Mobility （Total 6 Point）		Left	3				
		Right	3				
Check 5: Upper Extremity Stability & Strength （Total 4 Point）			4				
Check 6: Hip Mobility （Total 8 Point）	Flex/ ER	Left	1				
		Right	1				
	Flex/ IR	Left	1				
		Right	1				
	Ext/ ER	Left	1				
		Right	1				
	Ext/ IR	Left	1				
		Right	1				
Check 7: Hip and Spinal Mobility （Total 6 Point）		Front	3				
		Back	3				
Check 8: Trunk and Lower Extremity Mobility & Stability （Total 2 Point）		Left	1				
		Right	1				
Check 9: Trunk Strength （Total 3 Point）			4				
Check 10: Lower Extremity Strength (Total 8 Point)		Left	4				
		Right	4				
Check 11: Ankle Mobility （Total 2 Point）		Left	1				
		Right	1				
Total Score (50)			50	0	0	0	0
