# Supplementary material for: Validity of the KOJI AWARENESS self-screening test for body movement and comparison with functional movement screening
Source: PLoS One. 2022 Dec 30;17(12):e0277167. doi: 10.1371/journal.pone.0277167 (PMC9803145; doi:10.1371/journal.pone.0277167)
Supplement: S2 Appendix — (PDF) [file pone.0277167.s003.pdf]

S2 Appendix **Scoring Criteria**

Components	→ Set Up →	→ Movement →	criteria
①Neck Mobility	stand in front of mirror	bring the chin towards chest	1 point: he/she can touch the chest with chin<br>0 point: he/she can NOT touch the chest with chin
	stand in front of mirror with hands on waist, set elbows in a straight line when viewed from the top	tilt head to one side	1 point: he/she can tilt the mid line of face parallel to the upper arm<br>0 point: he/she can NOT tilt the mid line of face parallel to the upper arm
	stand side way in front of mirror with hands on waist, set elbows in a straight line when viewed from the top	rotate head without moving the shoulder	1 point: he/she can tilt the mid line of face parallel to the shoulder<br>0 point: he/she can NOT tilt the mid line of face parallel to the shoulder
	lie on elbows and knees with finger tips at 4 foot lengths away from wall	look up to the wall	1 point: he/she can see 2-elbow height mark on wall without difficulty<br>0 point: he/she can NOT see 2-elbow height mark on wall without difficulty
②Shoulder Mobility	stand with a back of hand on lower back	reach towards the opposite shoulder blade	touch inferior angle of the blade
③Shoulder Blade (Scapular) Mobility	stand in front of mirror with finger tips holding opposite earlobe	move the arm around the head and back	1 point: he/she can make the Arch of the arm go around the head without head tilt<br>0 point: he/she can NOT make the Arch of the arm go around the head without head tilt
④Thoracic Spine Mobility (3 levels)	sit 2 knuckles away from wall with knees together, hands on shoulders with elbows in a straight line when viewed from the top	rotate body to reach towards wall with elbow	1 point: he/she can touch wall with elbow while knees are together<br>0 point: he/she can NOT touch wall with elbow while knees are together
	sit 2 knuckles away from wall with knees together	rotate body to reach towards wall with opposite hand	1 point: he/she can touch wall with hand by the shoulder, while knees are together<br>0 point: he/she can NOT touch wall with hand by the shoulder, while knees are together
	sit 2 knuckles away from wall with knees together, hands on opposite shoulders, set elbows at shoulder height	rotate body to reach towards wall	1 point: he/she can touch wall with upper arm between shoulder and elbow, while knees are together<br>0 point: he/she can NOT touch wall with upper arm between shoulder and elbow, while knees are together
⑤Upper Extremity Stability & Strength (4 levels)	create front hand-plank position on wall, feet at 4 foot lengths away from wall, hands shoulder width at level of eyes	hold plank position for 10 seconds	1 point: he/she can hold position for 10 seconds while head, pelvis, ankles are in line<br>0 point: he/she can NOT hold position for 10 seconds while head, pelvis, ankles are in line
	create front hand-plank position on floor with bent knees	hold plank position for 10 seconds	
	create front hand-plank position on floor	hold plank position in different conditions	
	start with front hand-plank position, then side hand-plank followed by opposite side.	hold plank position for 5, 3 and 3 seconds respectively	1 point: he/she can hold each position while head, pelvis, ankles are in line<br>0 point: he/she can NOT hold each position while head, pelvis, ankles are in line
⑥Hip Mobility	stand in front of wall, 1 foot & 1 knuckle away, keep a knee on wall with the thigh parallel to the floor	rotate leg to inside, touch ankle	1 point: he/she can touch medial malleolus without tilting torso<br>0 point: he/she can NOT touch medial malleolus without tilting torso
	stand in front of wall, 1 foot & 1 knuckle away, keep a knee on wall with the thigh parallel to the floor	rotate leg to outside, touch ankle	1 point: he/she can touch lateral malleolus without tilting torso<br>0 point: he/she can NOT touch lateral malleolus without tilting torso
	bend knee towards buttocks while standing on one leg, keep knees together	rotate leg to inside, touch ankle	1 point: he/she can touch medial malleolus without tilting torso<br>0 point: he/she can NOT touch medial malleolus without tilting torso
	bend knee towards buttocks while standing on one leg, keep knees together	rotate leg to outside, touch ankle	1 point: he/she can touch lateral malleolus without tilting torso<br>0 point: he/she can NOT touch lateral malleolus without tilting torso
⑦Hip and Spine Mobility (3 levels)	stand with feet shoulder width, mark at knuckle length from ankle	bend over to reach the mark	1 point: he/she can touch one knuckle above ankle<br>0 point: he/she can NOT touch one knuckle above ankle
	stand with feet shoulder width	bend over to reach ankle joint	1 point: he/she can touch ankle joint<br>0 point: he/she can NOT touch ankle joint
	stand with feet shoulder width	bend over to reach toes	1 point: he/she can touch toes<br>0 point: he/she can NOT touch toes
	stand 1 foot length away from wall with back facing wall	touch wall with hands over head	1 point: he/she can ttouch wall from 1 foot length away<br>0 point: he/she can NOT touch wall from 1 foot length away
	stand 2 foot lengthes away from wall with back facing wall	touch wall with hands over head	1 point: he/she can touch wall from 2 feet length away<br>0 point: he/she can NOT touch wall from 2 feet length away
	stand 2 foot and 1 knuckle lengthes away from wall with back facing wall	touch wall with hands over head	1 point: he/she can touch wall from 2 feet and 1 knuckle length away<br>0 point: he/she can NOT touch wall from 2 feet and 1 knuckle length away
⑧Upper and Lower Extremity Mobility & Stability	stand with feet shoulder width	bring one side of elbow and knee together	1 point: he/she can hold elbow and knee together for 5 seconds without tilting torso<br>0 point: he/she can NOT hold elbow and knee together for 5 seconds without tilting torso
	stand with back against wall, heels 1 knuckle away from wall	bring one side of elbow and knee together	1 point: he/she can touch elbow to the knee while back of head and opposite upper and lower back stay on the wall<br>0 point: he/she can NOT touch elbow to the knee while back of head and opposite upper and lower back stay on the wall
⑨Mid-section Stability & Strength (4 levels)	lie on back on floor with knees bent, arms straight by torso	bring shoudler blades away from the floor	"1 point: he/she can hold for 5 seconds while shoulder blades are away from floor<br>0 point: he/she can NOT hold for 5 seconds while shoulder blades are away from floor
	lie on back on floor with knees straight, arms straight by torso	bring shoulder blades away from the floor	
	lie on back on floor with knees straight, hands on opposite shoudlers	bring shoulder blades away from the floor	
	lie on back on floor with knees straight, hands on back of head	bring shoulder blades away from the floor	
⑩Lower Extremity Strength (4 levels)	Sit on half kneeling position, hands on front knee	stand up and down using hands	1 point: he/she can stand up and down without losing control<br>0 point: he/she can NOT stand up and down without losing control
	Sit on half kneeling position, hands on waist	stand up and down	
	Sit on chair with one leg off floor, hands on opposite shoulders	stand up and down with one leg	
	Sit on chair with legs crossed, hands on opposite shoulders	stand up and down with legs crossed	
⑪Ankle Mobility	toe 1 knuckle away from wall while in half kneeling	bring knee to wall	1 point: he/she can touch wall by knee without lifting heel<br>0 point: he/she can NOT touch wall by knee without lifting heel
